# Supplementary material for: An evaluation of the readability and visual appearance of online patient resources for fibroadenoma
Source: PLoS One. 2022 Nov 18;17(11):e0277823. doi: 10.1371/journal.pone.0277823 (PMC9674151; doi:10.1371/journal.pone.0277823)
Supplement: S1 File — (DOCX) [file pone.0277823.s001.docx]

Supporting Information

S1 Table. Top 10 websites identified using search terms with Google^TM^ Bing^TM^ and Yahoo^TM^ search engines.

| Search term | Bing^TM^ | Google^TM^ | Yahoo^TM^ |
| --- | --- | --- | --- |
| “Fibroadenoma” | 1. Breast Cancer Organisation  2. Breast Cancer Now  3. Wikipedia  4. The Women’s Hospital  5. National Health Service  6. Buoy Health  7. Cleveland Clinic  8. Healthline  9. HCA Healthcare  10. My Breast My Health | 1. Mayo Clinic  2. Breast Cancer Now  3. American Cancer Society  4. WebMD Cancer Center  5. Healthline  6. Breast Cancer Organisation  7. The Women’s Hospital  8. National Health Service  9. Cleveland Clinic  10. Medical News Today | 1. Breast Cancer Organisation  2. Breast Cancer Now  3. Wikipedia  4. Buoy Health  5. WebMD Cancer Center  6. My Breast My Health  7. gp notebook  8. Teach Me Surgery  9. Cleveland Clinic  10. Healthline |
| “breast lumps” | 1. National Health Service  2. Patient info  3. Mayo Clinic  4. Very Well Health  5. Bupa UK  6. Net Doctor  7. Healthline  8. Medical News Today  9. WebMD Cancer Center  10. Medicine.net | 1. Breast Cancer Now  2. Cancer Research UK  3. National Health Service  4. Patient Info  5. Mayo Clinic  6. Bupa UK  7. Very Well Health  8. Cleveland Clinic  9. Healthline  10. Medical News Today | 1. National Health Service  2. Medical News Today  3. Breast Cancer Now  4. Mayo Clinic  5. WebMD Cancer Center  6. Radiology info. org  7. Stony Brook Cancer Center  8. Bupa UK  9. Cleveland Clinic  10. Healthline |
| “non-cancerous breast lumps” | 1. American Cancer Society  2. John Hopkins Medicine  3. Nathan T Thomas MD  4. Bupa UK  5. Memorial Sloan Kettering Cancer Center  6. Breast Cancer Now  7. Patient.info  8. ICliniq  9. Healthline  10. Very Well Health | 1. John Hopkins Medicine  2. National Health Service  3. American Cancer Society  4. Bupa UK  5. Breast Cancer Now  6. Mayo Clinic  7. Memorial Sloan Kettering Cancer Center  8. Cleveland Clinic  9. WebMD Cancer Center  10. Stony Brook Cancer Center | 1. Breast Cancer Now  2. Cancer Research UK  3. American Cancer Society  4. Very Well Health  5. Nathan T Thomas MD  6. Memorial Sloan Kettering Cancer Center  7. Shape.com  8. Cleveland Clinic  9. Patient.info  10. ICliniq |
| “benign breast lumps” | 1. Bupa UK  2. John Hopkins Medicine  3. Cleveland Clinic  4. The London Clinic  5. National Health Service  6. Breast Cancer Organisation  7. Patient.info  8. Net Doctor  9. Teach Me Surgery  10. Total Health | 1. John Hopkins Medicine  2. Bupa UK  3. Breast Cancer Now  4. National Health Service  5. Mayo Clinic  6. WebMD Cancer Center  7. American Cancer Society  8. Cleveland Clinic  9. Stony Brook Cancer Center  10. Breast Cancer Organisation | 1. Breast Cancer Now  2. Cancer Research UK  3. Bupa UK  4. John Hopkins Medicine  5. The London Clinic  6. Cleveland Clinic  7. National Health Service  8. Breast Cancer Organisation  9. Patient.info  10. Teach Me Surgery |
| “benign breast lesions” | 1. Breast Cancer Organisation  2. Cleveland Clinic  3. American Cancer Society  4. Bupa UK  5. John Hopkins Medicine  6. Very Well Health  7. Medanta  8. Teach Me Surgery  9. Specialist Breast Cancer Surgery  10. Breast Cancer Hub | 1. John Hopkins Medicine  2. Cleveland Clinic  3. Breast Cancer Specialist  4. Breast Cancer Organisation  5. American Cancer Society  6. Breast Cancer Now  7. Family Doctor  8. Moffitt Cancer Center  9. Bupa UK  10. Mayo Clinic | 1. Cancer Research UK  2. Cleveland Clinic  3. Breast Cancer Organisation  4. American Cancer Society  5. Breast Cancer Hub  6. Komen.org  7. Teach Me Surgery  8. Breast Cancer Now  9. iheartpathology  10. John Hopkins Medical |

S2 Table. Visual assessment of the 39 identified websites.


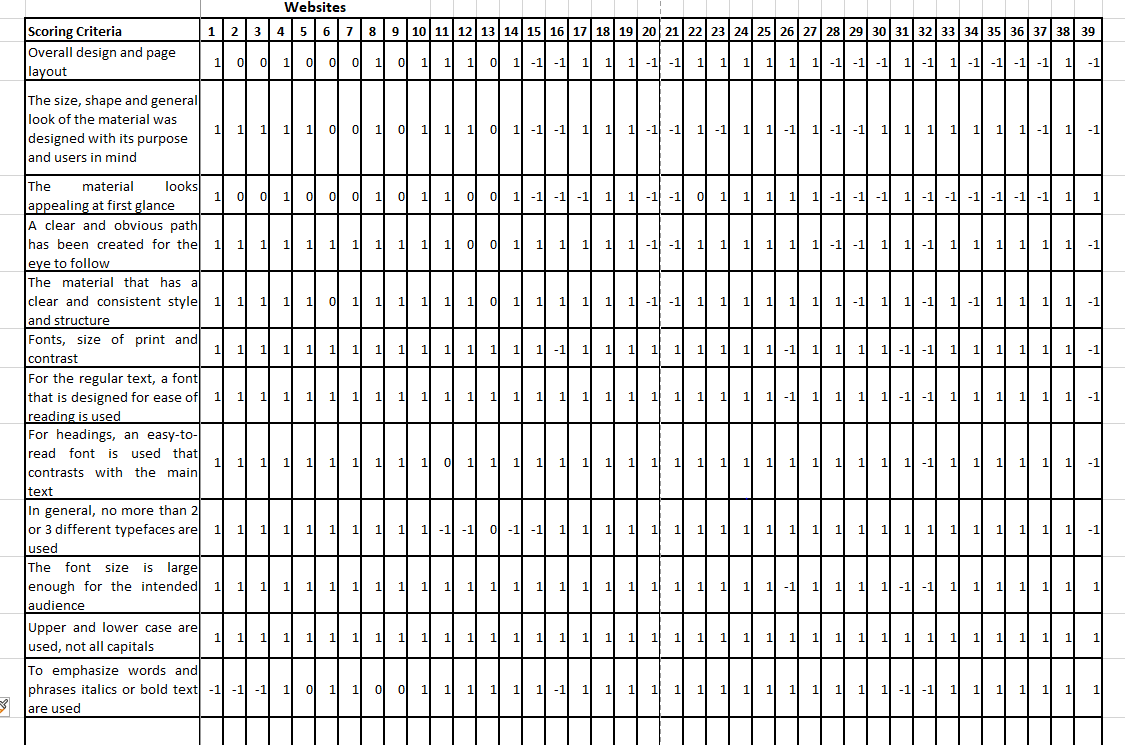

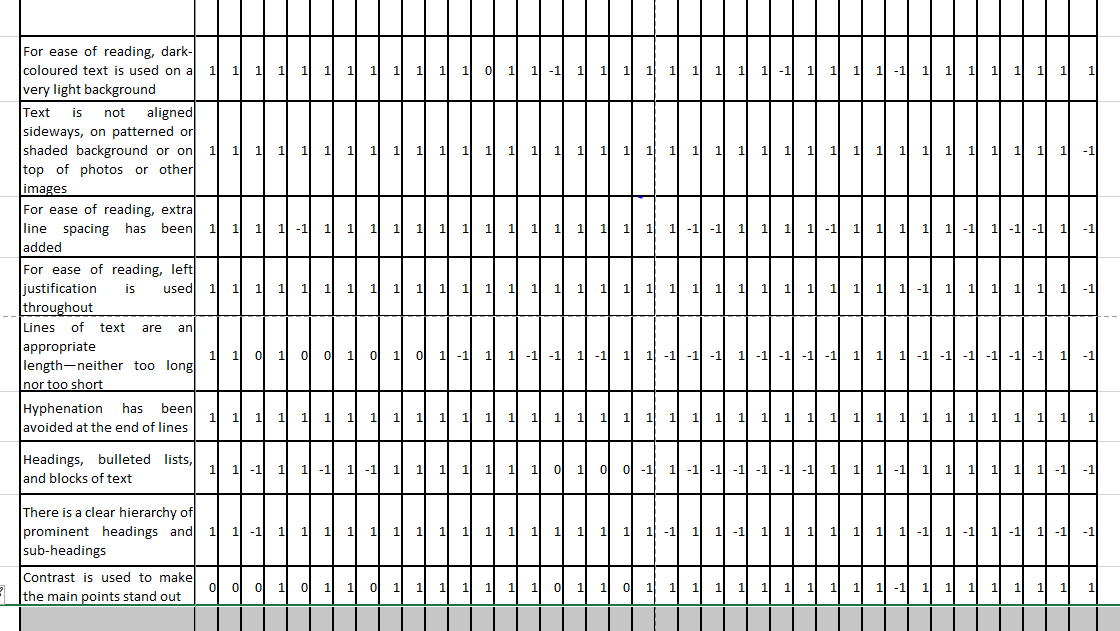

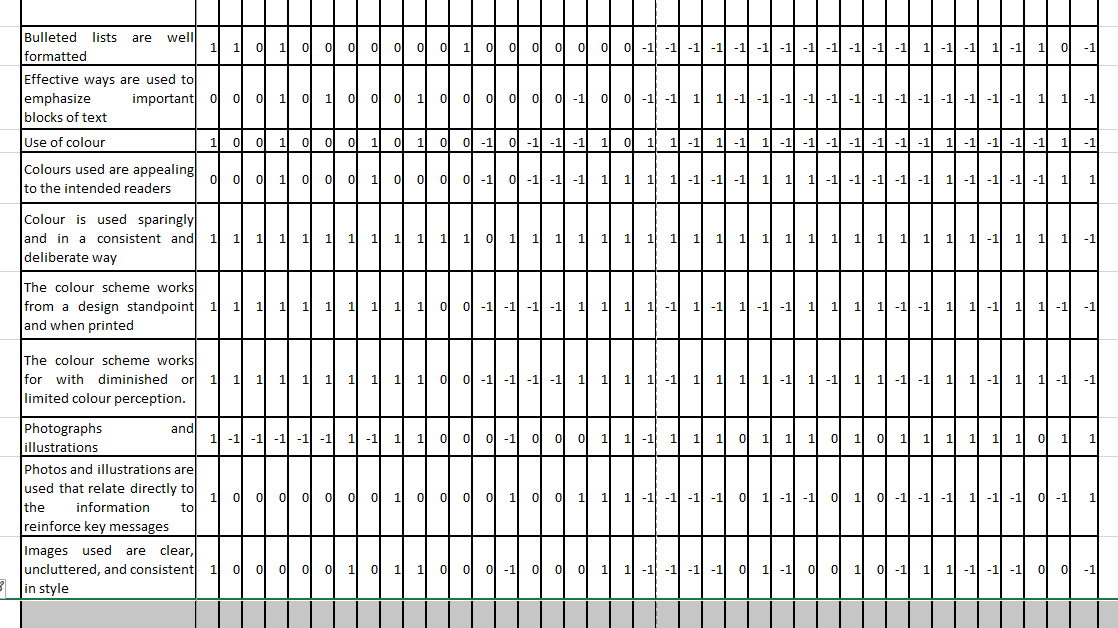

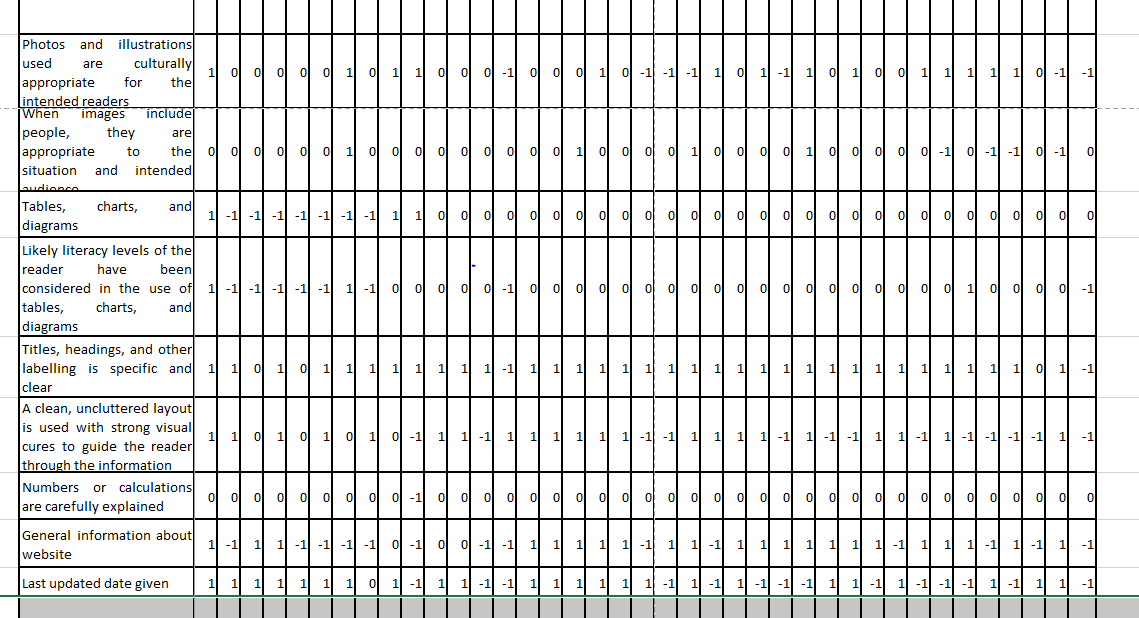

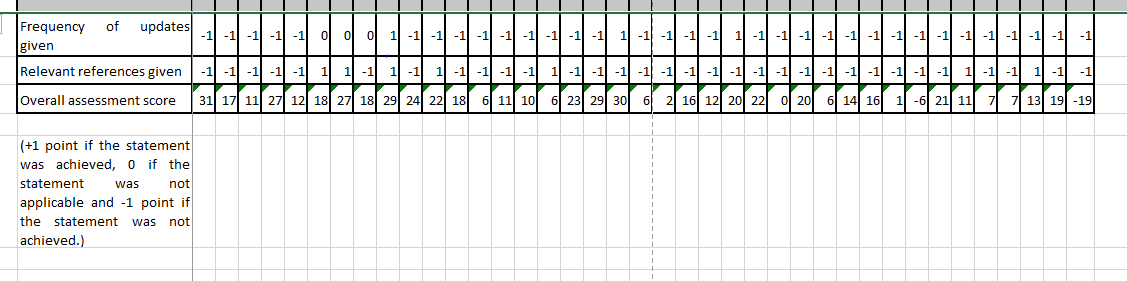


S3 Table. Post-hoc analysis to identify differences between website median readability scores

| **Pairwise Comparisons of websitecategory** | | | |
| --- | --- | --- | --- |
| Sample 1-Sample 2 | Test Statistic | Sig. | Adj. Sig.^a^ |
| Medanta-Cancer Research UK | .160 | .689 | 1.000 |
| Medanta-HCA Healthcare | .400 | .527 | 1.000 |
| Medanta-American Cancer Society | .160 | .689 | 1.000 |
| Medanta-NHS | .240 | .624 | 1.000 |
| Medanta-Moffitt | .400 | .527 | 1.000 |
| Medanta-WebMD | .229 | .633 | 1.000 |
| Medanta-Memorial Sloan Kettering Cancer Center | .400 | .527 | 1.000 |
| Medanta-Breast cancer Now | .182 | .670 | 1.000 |
| Medanta-Patient info | .172 | .679 | 1.000 |
| Medanta-Bupa | .240 | .624 | 1.000 |
| Medanta-Family Doctor | .400 | .527 | 1.000 |
| Medanta-My Breast My Health | .267 | .606 | 1.000 |
| Medanta-Total Health | .400 | .527 | 1.000 |
| Medanta-The Women's Hospital | .229 | .633 | 1.000 |
| Medanta-The London Clinic | .400 | .527 | 1.000 |
| Medanta-Buoy Health | .178 | .673 | 1.000 |
| Medanta-John Hopkins Medicine | .160 | .689 | 1.000 |
| Medanta-Net Doctor | .134 | .714 | 1.000 |
| Medanta-iqclinic | .160 | .689 | 1.000 |
| Medanta-Komen | .222 | .637 | 1.000 |
| Medanta-Breast cancer org | .182 | .670 | 1.000 |
| Medanta-Mayo Clinic | .172 | .679 | 1.000 |
| Medanta-Cleveland clinic | .240 | .624 | 1.000 |
| Medanta-Healthline | .185 | .667 | 1.000 |
| Medanta-Up to date | .400 | .527 | 1.000 |
| Medanta-Very Well Health | 5.556 | .018 | 1.000 |
| Medanta-Medical News Today | 1.963 | .161 | 1.000 |
| Medanta-Shape | 6.563 | .010 | 1.000 |
| Medanta-Stony Brook Medical Center | 10.000 | .002 | 1.000 |
| Medanta-Wikipedia | 5.304 | .021 | 1.000 |
| Medanta-iheartpathology | .400 | .527 | 1.000 |
| Medanta-Medicine.net | 5.380 | .020 | 1.000 |
| Medanta-Breast Cancer Hub | .400 | .527 | 1.000 |
| Medanta-Gp notebook | 5.714 | .017 | 1.000 |
| Medanta-Radiology info | 5.304 | .021 | 1.000 |
| Medanta-Teach me surgery | 10.000 | .002 | 1.000 |
| Medanta-Specialist Breast Cancer Surgery | 10.000 | .002 | 1.000 |
| Medanta-Nathan T Thomas | 3.600 | .058 | 1.000 |
| Cancer Research UK-HCA Healthcare | .041 | .840 | 1.000 |
| Cancer Research UK-American Cancer Society | .400 | .527 | 1.000 |
| Cancer Research UK-NHS | .218 | .641 | 1.000 |
| Cancer Research UK-Moffitt | .361 | .548 | 1.000 |
| Cancer Research UK-WebMD | .210 | .646 | 1.000 |
| Cancer Research UK-Memorial Sloan Kettering Cancer Center | .361 | .548 | 1.000 |
| Cancer Research UK-Breast cancer Now | 1.120 | .290 | 1.000 |
| Cancer Research UK-Patient info | 1.333 | .248 | 1.000 |
| Cancer Research UK-Bupa | 1.138 | .286 | 1.000 |
| Cancer Research UK-Family Doctor | .361 | .548 | 1.000 |
| Cancer Research UK-My Breast My Health | 1.373 | .241 | 1.000 |
| Cancer Research UK-Total Health | .361 | .548 | 1.000 |
| Cancer Research UK-The Women's Hospital | 2.497 | .114 | 1.000 |
| Cancer Research UK-The London Clinic | .361 | .548 | 1.000 |
| Cancer Research UK-Buoy Health | 2.700 | .100 | 1.000 |
| Cancer Research UK-John Hopkins Medicine | 3.600 | .058 | 1.000 |
| Cancer Research UK-Net Doctor | 2.400 | .121 | 1.000 |
| Cancer Research UK-iqclinic | 3.600 | .058 | 1.000 |
| Cancer Research UK-Komen | 2.341 | .126 | 1.000 |
| Cancer Research UK-Breast cancer org | 2.520 | .112 | 1.000 |
| Cancer Research UK-Mayo Clinic | 3.000 | .083 | 1.000 |
| Cancer Research UK-Cleveland clinic | 2.779 | .095 | 1.000 |
| Cancer Research UK-Healthline | 8.822 | .003 | 1.000 |
| Cancer Research UK-Up to date | 2.564 | .109 | 1.000 |
| Cancer Research UK-Very Well Health | 9.876 | .002 | 1.000 |
| Cancer Research UK-Medical News Today | 3.600 | .058 | 1.000 |
| Cancer Research UK-Shape | 5.400 | .020 | 1.000 |
| Cancer Research UK-Stony Brook Medical Center | 6.771 | .009 | 1.000 |
| Cancer Research UK-Wikipedia | 22.680 | <.001 | .001 |
| Cancer Research UK-iheartpathology | 6.771 | .009 | 1.000 |
| Cancer Research UK-Medicine.net | 14.700 | <.001 | .093 |
| Cancer Research UK-Breast Cancer Hub | 6.771 | .009 | 1.000 |
| Cancer Research UK-Gp notebook | 30.306 | <.001 | .000 |
| Cancer Research UK-Radiology info | 26.444 | <.001 | .000 |
| Cancer Research UK-Teach me surgery | 6.771 | .009 | 1.000 |
| Cancer Research UK-Specialist Breast Cancer Surgery | 6.771 | .009 | 1.000 |
| Cancer Research UK-Nathan T Thomas | 6.771 | .009 | 1.000 |
| HCA Healthcare-American Cancer Society | .160 | .689 | 1.000 |
| HCA Healthcare-NHS | .240 | .624 | 1.000 |
| HCA Healthcare-Moffitt | .400 | .527 | 1.000 |
| HCA Healthcare-WebMD | .229 | .633 | 1.000 |
| HCA Healthcare-Memorial Sloan Kettering Cancer Center | .400 | .527 | 1.000 |
| HCA Healthcare-Breast cancer Now | .182 | .670 | 1.000 |
| HCA Healthcare-Patient info | .172 | .679 | 1.000 |
| HCA Healthcare-Bupa | .240 | .624 | 1.000 |
| HCA Healthcare-Family Doctor | .400 | .527 | 1.000 |
| HCA Healthcare-My Breast My Health | .267 | .606 | 1.000 |
| HCA Healthcare-Total Health | .400 | .527 | 1.000 |
| HCA Healthcare-The Women's Hospital | .229 | .633 | 1.000 |
| HCA Healthcare-The London Clinic | .400 | .527 | 1.000 |
| HCA Healthcare-Buoy Health | .178 | .673 | 1.000 |
| HCA Healthcare-John Hopkins Medicine | .160 | .689 | 1.000 |
| HCA Healthcare-Net Doctor | .134 | .714 | 1.000 |
| HCA Healthcare-iqclinic | .160 | .689 | 1.000 |
| HCA Healthcare-Komen | .222 | .637 | 1.000 |
| HCA Healthcare-Breast cancer org | .182 | .670 | 1.000 |
| HCA Healthcare-Mayo Clinic | 1.906 | .167 | 1.000 |
| HCA Healthcare-Cleveland clinic | .240 | .624 | 1.000 |
| HCA Healthcare-Healthline | 1.852 | .174 | 1.000 |
| HCA Healthcare-Up to date | .400 | .527 | 1.000 |
| HCA Healthcare-Very Well Health | 2.000 | .157 | 1.000 |
| HCA Healthcare-Medical News Today | 1.963 | .161 | 1.000 |
| HCA Healthcare-Shape | 2.143 | .143 | 1.000 |
| HCA Healthcare-Stony Brook Medical Center | 3.600 | .058 | 1.000 |
| HCA Healthcare-Wikipedia | 5.304 | .021 | 1.000 |
| HCA Healthcare-iheartpathology | .400 | .527 | 1.000 |
| HCA Healthcare-Medicine.net | 5.380 | .020 | 1.000 |
| HCA Healthcare-Breast Cancer Hub | 3.600 | .058 | 1.000 |
| HCA Healthcare-Gp notebook | 5.714 | .017 | 1.000 |
| HCA Healthcare-Radiology info | 5.304 | .021 | 1.000 |
| HCA Healthcare-Teach me surgery | 3.600 | .058 | 1.000 |
| HCA Healthcare-Specialist Breast Cancer Surgery | 3.600 | .058 | 1.000 |
| HCA Healthcare-Nathan T Thomas | 3.600 | .058 | 1.000 |
| American Cancer Society-NHS | .218 | .641 | 1.000 |
| American Cancer Society-Moffitt | .361 | .548 | 1.000 |
| American Cancer Society-WebMD | .210 | .646 | 1.000 |
| American Cancer Society-Memorial Sloan Kettering Cancer Center | .361 | .548 | 1.000 |
| American Cancer Society-Breast cancer Now | .280 | .597 | 1.000 |
| American Cancer Society-Patient info | .333 | .564 | 1.000 |
| American Cancer Society-Bupa | .218 | .641 | 1.000 |
| American Cancer Society-Family Doctor | .361 | .548 | 1.000 |
| American Cancer Society-My Breast My Health | .238 | .625 | 1.000 |
| American Cancer Society-Total Health | .361 | .548 | 1.000 |
| American Cancer Society-The Women's Hospital | .210 | .646 | 1.000 |
| American Cancer Society-The London Clinic | .361 | .548 | 1.000 |
| American Cancer Society-Buoy Health | .300 | .584 | 1.000 |
| American Cancer Society-John Hopkins Medicine | .400 | .527 | 1.000 |
| American Cancer Society-Net Doctor | .600 | .439 | 1.000 |
| American Cancer Society-iqclinic | .400 | .527 | 1.000 |
| American Cancer Society-Komen | .207 | .649 | 1.000 |
| American Cancer Society-Breast cancer org | .280 | .597 | 1.000 |
| American Cancer Society-Mayo Clinic | .333 | .564 | 1.000 |
| American Cancer Society-Cleveland clinic | .218 | .641 | 1.000 |
| American Cancer Society-Healthline | 2.400 | .121 | 1.000 |
| American Cancer Society-Up to date | .361 | .548 | 1.000 |
| American Cancer Society-Very Well Health | 2.341 | .126 | 1.000 |
| American Cancer Society-Medical News Today | .400 | .527 | 1.000 |
| American Cancer Society-Shape | 5.400 | .020 | 1.000 |
| American Cancer Society-Stony Brook Medical Center | 6.771 | .009 | 1.000 |
| American Cancer Society-Wikipedia | 10.080 | .001 | 1.000 |
| American Cancer Society-iheartpathology | .361 | .548 | 1.000 |
| American Cancer Society-Medicine.net | 4.800 | .028 | 1.000 |
| American Cancer Society-Breast Cancer Hub | 2.564 | .109 | 1.000 |
| American Cancer Society-Gp notebook | 19.217 | <.001 | .009 |
| American Cancer Society-Radiology info | 13.720 | <.001 | .157 |
| American Cancer Society-Teach me surgery | 6.771 | .009 | 1.000 |
| American Cancer Society-Specialist Breast Cancer Surgery | 6.771 | .009 | 1.000 |
| American Cancer Society-Nathan T Thomas | 6.771 | .009 | 1.000 |
| NHS-Moffitt | .240 | .624 | 1.000 |
| NHS-WebMD | .069 | .793 | 1.000 |
| NHS-Memorial Sloan Kettering Cancer Center | .240 | .624 | 1.000 |
| NHS-Breast cancer Now | .427 | .514 | 1.000 |
| NHS-Patient info | 1.516 | .218 | 1.000 |
| NHS-Bupa | .720 | .396 | 1.000 |
| NHS-Family Doctor | .240 | .624 | 1.000 |
| NHS-My Breast My Health | .960 | .327 | 1.000 |
| NHS-Total Health | .240 | .624 | 1.000 |
| NHS-The Women's Hospital | 3.360 | .067 | 1.000 |
| NHS-The London Clinic | .240 | .624 | 1.000 |
| NHS-Buoy Health | 1.385 | .239 | 1.000 |
| NHS-John Hopkins Medicine | 1.779 | .182 | 1.000 |
| NHS-Net Doctor | .732 | .392 | 1.000 |
| NHS-iqclinic | 6.421 | .011 | 1.000 |
| NHS-Komen | 5.040 | .025 | 1.000 |
| NHS-Breast cancer org | 4.507 | .034 | 1.000 |
| NHS-Mayo Clinic | 5.357 | .021 | 1.000 |
| NHS-Cleveland clinic | 3.920 | .048 | 1.000 |
| NHS-Healthline | 6.496 | .011 | 1.000 |
| NHS-Up to date | 2.160 | .142 | 1.000 |
| NHS-Very Well Health | 7.529 | .006 | 1.000 |
| NHS-Medical News Today | 6.421 | .011 | 1.000 |
| NHS-Shape | 9.619 | .002 | 1.000 |
| NHS-Stony Brook Medical Center | 6.000 | .014 | 1.000 |
| NHS-Wikipedia | 15.385 | <.001 | .065 |
| NHS-iheartpathology | 6.000 | .014 | 1.000 |
| NHS-Medicine.net | 13.888 | <.001 | .144 |
| NHS-Breast Cancer Hub | 6.000 | .014 | 1.000 |
| NHS-Gp notebook | 19.817 | <.001 | .006 |
| NHS-Radiology info | 16.670 | <.001 | .033 |
| NHS-Teach me surgery | 6.000 | .014 | 1.000 |
| NHS-Specialist Breast Cancer Surgery | 6.000 | .014 | 1.000 |
| NHS-Nathan T Thomas | 6.000 | .014 | 1.000 |
| Moffitt-WebMD | .229 | .633 | 1.000 |
| Moffitt-Memorial Sloan Kettering Cancer Center | .400 | .527 | 1.000 |
| Moffitt-Breast cancer Now | .182 | .670 | 1.000 |
| Moffitt-Patient info | .172 | .679 | 1.000 |
| Moffitt-Bupa | .240 | .624 | 1.000 |
| Moffitt-Family Doctor | .400 | .527 | 1.000 |
| Moffitt-My Breast My Health | .267 | .606 | 1.000 |
| Moffitt-Total Health | .400 | .527 | 1.000 |
| Moffitt-The Women's Hospital | .229 | .633 | 1.000 |
| Moffitt-The London Clinic | .400 | .527 | 1.000 |
| Moffitt-Buoy Health | .178 | .673 | 1.000 |
| Moffitt-John Hopkins Medicine | .160 | .689 | 1.000 |
| Moffitt-Net Doctor | .134 | .714 | 1.000 |
| Moffitt-iqclinic | .160 | .689 | 1.000 |
| Moffitt-Komen | .222 | .637 | 1.000 |
| Moffitt-Breast cancer org | .182 | .670 | 1.000 |
| Moffitt-Mayo Clinic | .172 | .679 | 1.000 |
| Moffitt-Cleveland clinic | .240 | .624 | 1.000 |
| Moffitt-Healthline | .185 | .667 | 1.000 |
| Moffitt-Up to date | .400 | .527 | 1.000 |
| Moffitt-Very Well Health | .222 | .637 | 1.000 |
| Moffitt-Medical News Today | .160 | .689 | 1.000 |
| Moffitt-Shape | .134 | .714 | 1.000 |
| Moffitt-Stony Brook Medical Center | 3.600 | .058 | 1.000 |
| Moffitt-Wikipedia | 1.862 | .172 | 1.000 |
| Moffitt-iheartpathology | .400 | .527 | 1.000 |
| Moffitt-Medicine.net | 1.879 | .170 | 1.000 |
| Moffitt-Breast Cancer Hub | .400 | .527 | 1.000 |
| Moffitt-Gp notebook | 2.057 | .151 | 1.000 |
| Moffitt-Radiology info | 5.304 | .021 | 1.000 |
| Moffitt-Teach me surgery | 3.600 | .058 | 1.000 |
| Moffitt-Specialist Breast Cancer Surgery | 3.600 | .058 | 1.000 |
| Moffitt-Nathan T Thomas | 3.600 | .058 | 1.000 |
| WebMD-Memorial Sloan Kettering Cancer Center | .229 | .633 | 1.000 |
| WebMD-Breast cancer Now | .325 | .568 | 1.000 |
| WebMD-Patient info | .375 | .540 | 1.000 |
| WebMD-Bupa | .231 | .631 | 1.000 |
| WebMD-Family Doctor | .229 | .633 | 1.000 |
| WebMD-My Breast My Health | .857 | .355 | 1.000 |
| WebMD-Total Health | .229 | .633 | 1.000 |
| WebMD-The Women's Hospital | 1.429 | .232 | 1.000 |
| WebMD-The London Clinic | .229 | .633 | 1.000 |
| WebMD-Buoy Health | 1.101 | .294 | 1.000 |
| WebMD-John Hopkins Medicine | 1.497 | .221 | 1.000 |
| WebMD-Net Doctor | .635 | .425 | 1.000 |
| WebMD-iqclinic | 1.497 | .221 | 1.000 |
| WebMD-Komen | 1.270 | .260 | 1.000 |
| WebMD-Breast cancer org | 1.023 | .312 | 1.000 |
| WebMD-Mayo Clinic | 1.233 | .267 | 1.000 |
| WebMD-Cleveland clinic | 1.714 | .190 | 1.000 |
| WebMD-Healthline | 2.822 | .093 | 1.000 |
| WebMD-Up to date | 2.057 | .151 | 1.000 |
| WebMD-Very Well Health | 4.114 | .043 | 1.000 |
| WebMD-Medical News Today | 1.497 | .221 | 1.000 |
| WebMD-Shape | 4.980 | .026 | 1.000 |
| WebMD-Stony Brook Medical Center | 5.714 | .017 | 1.000 |
| WebMD-Wikipedia | 12.086 | <.001 | .376 |
| WebMD-iheartpathology | 5.714 | .017 | 1.000 |
| WebMD-Medicine.net | 11.317 | <.001 | .569 |
| WebMD-Breast Cancer Hub | 5.714 | .017 | 1.000 |
| WebMD-Gp notebook | 25.200 | <.001 | .000 |
| WebMD-Radiology info | 13.367 | <.001 | .190 |
| WebMD-Teach me surgery | 5.714 | .017 | 1.000 |
| WebMD-Specialist Breast Cancer Surgery | 5.714 | .017 | 1.000 |
| WebMD-Nathan T Thomas | 5.714 | .017 | 1.000 |
| Memorial Sloan Kettering Cancer Center-Breast cancer Now | .182 | .670 | 1.000 |
| Memorial Sloan Kettering Cancer Center-Patient info | .172 | .679 | 1.000 |
| Memorial Sloan Kettering Cancer Center-Bupa | .240 | .624 | 1.000 |
| Memorial Sloan Kettering Cancer Center-Family Doctor | .400 | .527 | 1.000 |
| Memorial Sloan Kettering Cancer Center-My Breast My Health | .267 | .606 | 1.000 |
| Memorial Sloan Kettering Cancer Center-Total Health | .400 | .527 | 1.000 |
| Memorial Sloan Kettering Cancer Center-The Women's Hospital | .229 | .633 | 1.000 |
| Memorial Sloan Kettering Cancer Center-The London Clinic | .400 | .527 | 1.000 |
| Memorial Sloan Kettering Cancer Center-Buoy Health | .178 | .673 | 1.000 |
| Memorial Sloan Kettering Cancer Center-John Hopkins Medicine | .160 | .689 | 1.000 |
| Memorial Sloan Kettering Cancer Center-Net Doctor | .134 | .714 | 1.000 |
| Memorial Sloan Kettering Cancer Center-iqclinic | .160 | .689 | 1.000 |
| Memorial Sloan Kettering Cancer Center-Komen | .222 | .637 | 1.000 |
| Memorial Sloan Kettering Cancer Center-Breast cancer org | .182 | .670 | 1.000 |
| Memorial Sloan Kettering Cancer Center-Mayo Clinic | .172 | .679 | 1.000 |
| Memorial Sloan Kettering Cancer Center-Cleveland clinic | .240 | .624 | 1.000 |
| Memorial Sloan Kettering Cancer Center-Healthline | .185 | .667 | 1.000 |
| Memorial Sloan Kettering Cancer Center-Up to date | .400 | .527 | 1.000 |
| Memorial Sloan Kettering Cancer Center-Very Well Health | 2.000 | .157 | 1.000 |
| Memorial Sloan Kettering Cancer Center-Medical News Today | 1.963 | .161 | 1.000 |
| Memorial Sloan Kettering Cancer Center-Shape | 2.143 | .143 | 1.000 |
| Memorial Sloan Kettering Cancer Center-Stony Brook Medical Center | 3.600 | .058 | 1.000 |
| Memorial Sloan Kettering Cancer Center-Wikipedia | 5.304 | .021 | 1.000 |
| Memorial Sloan Kettering Cancer Center-iheartpathology | .400 | .527 | 1.000 |
| Memorial Sloan Kettering Cancer Center-Medicine.net | 5.380 | .020 | 1.000 |
| Memorial Sloan Kettering Cancer Center-Breast Cancer Hub | .400 | .527 | 1.000 |
| Memorial Sloan Kettering Cancer Center-Gp notebook | 5.714 | .017 | 1.000 |
| Memorial Sloan Kettering Cancer Center-Radiology info | 5.304 | .021 | 1.000 |
| Memorial Sloan Kettering Cancer Center-Teach me surgery | 3.600 | .058 | 1.000 |
| Memorial Sloan Kettering Cancer Center-Specialist Breast Cancer Surgery | 10.000 | .002 | 1.000 |
| Memorial Sloan Kettering Cancer Center-Nathan T Thomas | 3.600 | .058 | 1.000 |
| Breast cancer Now-Patient info | .000 | 1.000 | 1.000 |
| Breast cancer Now-Bupa | .027 | .870 | 1.000 |
| Breast cancer Now-Family Doctor | .182 | .670 | 1.000 |
| Breast cancer Now-My Breast My Health | .051 | .821 | 1.000 |
| Breast cancer Now-Total Health | .262 | .609 | 1.000 |
| Breast cancer Now-The Women's Hospital | .097 | .756 | 1.000 |
| Breast cancer Now-The London Clinic | .262 | .609 | 1.000 |
| Breast cancer Now-Buoy Health | .180 | .671 | 1.000 |
| Breast cancer Now-John Hopkins Medicine | 1.120 | .290 | 1.000 |
| Breast cancer Now-Net Doctor | .000 | 1.000 | 1.000 |
| Breast cancer Now-iqclinic | 1.120 | .290 | 1.000 |
| Breast cancer Now-Komen | 1.265 | .261 | 1.000 |
| Breast cancer Now-Breast cancer org | .640 | .424 | 1.000 |
| Breast cancer Now-Mayo Clinic | 1.920 | .166 | 1.000 |
| Breast cancer Now-Cleveland clinic | 1.707 | .191 | 1.000 |
| Breast cancer Now-Healthline | 8.353 | .004 | 1.000 |
| Breast cancer Now-Up to date | 2.103 | .147 | 1.000 |
| Breast cancer Now-Very Well Health | 5.559 | .018 | 1.000 |
| Breast cancer Now-Medical News Today | 2.520 | .112 | 1.000 |
| Breast cancer Now-Shape | 4.320 | .038 | 1.000 |
| Breast cancer Now-Stony Brook Medical Center | 5.704 | .017 | 1.000 |
| Breast cancer Now-Wikipedia | 27.040 | <.001 | .000 |
| Breast cancer Now-iheartpathology | 2.103 | .147 | 1.000 |
| Breast cancer Now-Medicine.net | 11.520 | <.001 | .510 |
| Breast cancer Now-Breast Cancer Hub | 5.704 | .017 | 1.000 |
| Breast cancer Now-Gp notebook | 42.023 | <.001 | .000 |
| Breast cancer Now-Radiology info | 27.040 | <.001 | .000 |
| Breast cancer Now-Teach me surgery | 5.704 | .017 | 1.000 |
| Breast cancer Now-Specialist Breast Cancer Surgery | 5.704 | .017 | 1.000 |
| Breast cancer Now-Nathan T Thomas | 5.704 | .017 | 1.000 |
| Patient info-Bupa | .010 | .921 | 1.000 |
| Patient info-Family Doctor | .305 | .581 | 1.000 |
| Patient info-My Breast My Health | .178 | .673 | 1.000 |
| Patient info-Total Health | .305 | .581 | 1.000 |
| Patient info-The Women's Hospital | .147 | .702 | 1.000 |
| Patient info-The London Clinic | .305 | .581 | 1.000 |
| Patient info-Buoy Health | .233 | .629 | 1.000 |
| Patient info-John Hopkins Medicine | 1.333 | .248 | 1.000 |
| Patient info-Net Doctor | .000 | 1.000 | 1.000 |
| Patient info-iqclinic | .333 | .564 | 1.000 |
| Patient info-Komen | 1.743 | .187 | 1.000 |
| Patient info-Breast cancer org | .853 | .356 | 1.000 |
| Patient info-Mayo Clinic | 2.400 | .121 | 1.000 |
| Patient info-Cleveland clinic | 2.183 | .140 | 1.000 |
| Patient info-Healthline | 2.690 | .101 | 1.000 |
| Patient info-Up to date | 2.307 | .129 | 1.000 |
| Patient info-Very Well Health | 3.209 | .073 | 1.000 |
| Patient info-Medical News Today | 3.000 | .083 | 1.000 |
| Patient info-Shape | 4.800 | .028 | 1.000 |
| Patient info-Stony Brook Medical Center | 6.176 | .013 | 1.000 |
| Patient info-Wikipedia | 21.333 | <.001 | .003 |
| Patient info-iheartpathology | 2.307 | .129 | 1.000 |
| Patient info-Medicine.net | 13.387 | <.001 | .188 |
| Patient info-Breast Cancer Hub | 6.176 | .013 | 1.000 |
| Patient info-Gp notebook | 28.725 | <.001 | .000 |
| Patient info-Radiology info | 21.333 | <.001 | .003 |
| Patient info-Teach me surgery | 6.176 | .013 | 1.000 |
| Patient info-Specialist Breast Cancer Surgery | 6.176 | .013 | 1.000 |
| Patient info-Nathan T Thomas | 6.176 | .013 | 1.000 |
| Bupa-Family Doctor | .240 | .624 | 1.000 |
| Bupa-My Breast My Health | .107 | .744 | 1.000 |
| Bupa-Total Health | .240 | .624 | 1.000 |
| Bupa-The Women's Hospital | .617 | .432 | 1.000 |
| Bupa-The London Clinic | .240 | .624 | 1.000 |
| Bupa-Buoy Health | .445 | .505 | 1.000 |
| Bupa-John Hopkins Medicine | .538 | .463 | 1.000 |
| Bupa-Net Doctor | .011 | .915 | 1.000 |
| Bupa-iqclinic | .538 | .463 | 1.000 |
| Bupa-Komen | 1.556 | .212 | 1.000 |
| Bupa-Breast cancer org | 1.307 | .253 | 1.000 |
| Bupa-Mayo Clinic | 1.516 | .218 | 1.000 |
| Bupa-Cleveland clinic | 2.000 | .157 | 1.000 |
| Bupa-Healthline | 3.739 | .053 | 1.000 |
| Bupa-Up to date | 2.160 | .142 | 1.000 |
| Bupa-Very Well Health | 5.040 | .025 | 1.000 |
| Bupa-Medical News Today | 1.779 | .182 | 1.000 |
| Bupa-Shape | 5.536 | .019 | 1.000 |
| Bupa-Stony Brook Medical Center | 6.000 | .014 | 1.000 |
| Bupa-Wikipedia | 15.385 | <.001 | .065 |
| Bupa-iheartpathology | 2.160 | .142 | 1.000 |
| Bupa-Medicine.net | 10.347 | .001 | .961 |
| Bupa-Breast Cancer Hub | 6.000 | .014 | 1.000 |
| Bupa-Gp notebook | 24.754 | <.001 | .000 |
| Bupa-Radiology info | 16.670 | <.001 | .033 |
| Bupa-Teach me surgery | 6.000 | .014 | 1.000 |
| Bupa-Specialist Breast Cancer Surgery | 6.000 | .014 | 1.000 |
| Bupa-Nathan T Thomas | 6.000 | .014 | 1.000 |
| Family Doctor-My Breast My Health | .267 | .606 | 1.000 |
| Family Doctor-Total Health | .400 | .527 | 1.000 |
| Family Doctor-The Women's Hospital | .229 | .633 | 1.000 |
| Family Doctor-The London Clinic | .400 | .527 | 1.000 |
| Family Doctor-Buoy Health | .178 | .673 | 1.000 |
| Family Doctor-John Hopkins Medicine | .160 | .689 | 1.000 |
| Family Doctor-Net Doctor | .134 | .714 | 1.000 |
| Family Doctor-iqclinic | .160 | .689 | 1.000 |
| Family Doctor-Komen | .222 | .637 | 1.000 |
| Family Doctor-Breast cancer org | .182 | .670 | 1.000 |
| Family Doctor-Mayo Clinic | .172 | .679 | 1.000 |
| Family Doctor-Cleveland clinic | .240 | .624 | 1.000 |
| Family Doctor-Healthline | .185 | .667 | 1.000 |
| Family Doctor-Up to date | .400 | .527 | 1.000 |
| Family Doctor-Very Well Health | .222 | .637 | 1.000 |
| Family Doctor-Medical News Today | .160 | .689 | 1.000 |
| Family Doctor-Shape | .134 | .714 | 1.000 |
| Family Doctor-Stony Brook Medical Center | .400 | .527 | 1.000 |
| Family Doctor-Wikipedia | .182 | .670 | 1.000 |
| Family Doctor-iheartpathology | .400 | .527 | 1.000 |
| Family Doctor-Medicine.net | 1.879 | .170 | 1.000 |
| Family Doctor-Breast Cancer Hub | .400 | .527 | 1.000 |
| Family Doctor-Gp notebook | 5.714 | .017 | 1.000 |
| Family Doctor-Radiology info | 5.304 | .021 | 1.000 |
| Family Doctor-Teach me surgery | 3.600 | .058 | 1.000 |
| Family Doctor-Specialist Breast Cancer Surgery | 3.600 | .058 | 1.000 |
| Family Doctor-Nathan T Thomas | .400 | .527 | 1.000 |
| My Breast My Health-Total Health | .267 | .606 | 1.000 |
| My Breast My Health-The Women's Hospital | .095 | .758 | 1.000 |
| My Breast My Health-The London Clinic | .267 | .606 | 1.000 |
| My Breast My Health-Buoy Health | .049 | .826 | 1.000 |
| My Breast My Health-John Hopkins Medicine | .038 | .845 | 1.000 |
| My Breast My Health-Net Doctor | .027 | .870 | 1.000 |
| My Breast My Health-iqclinic | .038 | .845 | 1.000 |
| My Breast My Health-Komen | .089 | .766 | 1.000 |
| My Breast My Health-Breast cancer org | .051 | .821 | 1.000 |
| My Breast My Health-Mayo Clinic | .711 | .399 | 1.000 |
| My Breast My Health-Cleveland clinic | .107 | .744 | 1.000 |
| My Breast My Health-Healthline | 1.616 | .204 | 1.000 |
| My Breast My Health-Up to date | .267 | .606 | 1.000 |
| My Breast My Health-Very Well Health | 4.356 | .037 | 1.000 |
| My Breast My Health-Medical News Today | .772 | .380 | 1.000 |
| My Breast My Health-Shape | 6.838 | .009 | 1.000 |
| My Breast My Health-Stony Brook Medical Center | 6.667 | .010 | 1.000 |
| My Breast My Health-Wikipedia | 6.666 | .010 | 1.000 |
| My Breast My Health-iheartpathology | .267 | .606 | 1.000 |
| My Breast My Health-Medicine.net | 6.984 | .008 | 1.000 |
| My Breast My Health-Breast Cancer Hub | 2.400 | .121 | 1.000 |
| My Breast My Health-Gp notebook | 16.095 | <.001 | .045 |
| My Breast My Health-Radiology info | 14.135 | <.001 | .126 |
| My Breast My Health-Teach me surgery | 6.667 | .010 | 1.000 |
| My Breast My Health-Specialist Breast Cancer Surgery | 6.667 | .010 | 1.000 |
| My Breast My Health-Nathan T Thomas | 6.667 | .010 | 1.000 |
| Total Health-The Women's Hospital | .229 | .633 | 1.000 |
| Total Health-The London Clinic | .400 | .527 | 1.000 |
| Total Health-Buoy Health | .178 | .673 | 1.000 |
| Total Health-John Hopkins Medicine | .160 | .689 | 1.000 |
| Total Health-Net Doctor | .134 | .714 | 1.000 |
| Total Health-iqclinic | .160 | .689 | 1.000 |
| Total Health-Komen | .222 | .637 | 1.000 |
| Total Health-Breast cancer org | .182 | .670 | 1.000 |
| Total Health-Mayo Clinic | .172 | .679 | 1.000 |
| Total Health-Cleveland clinic | .240 | .624 | 1.000 |
| Total Health-Healthline | .185 | .667 | 1.000 |
| Total Health-Up to date | .400 | .527 | 1.000 |
| Total Health-Very Well Health | 2.000 | .157 | 1.000 |
| Total Health-Medical News Today | 1.963 | .161 | 1.000 |
| Total Health-Shape | 2.143 | .143 | 1.000 |
| Total Health-Stony Brook Medical Center | 3.600 | .058 | 1.000 |
| Total Health-Wikipedia | 1.862 | .172 | 1.000 |
| Total Health-iheartpathology | .400 | .527 | 1.000 |
| Total Health-Medicine.net | 5.380 | .020 | 1.000 |
| Total Health-Breast Cancer Hub | .400 | .527 | 1.000 |
| Total Health-Gp notebook | 5.714 | .017 | 1.000 |
| Total Health-Radiology info | 5.304 | .021 | 1.000 |
| Total Health-Teach me surgery | 3.600 | .058 | 1.000 |
| Total Health-Specialist Breast Cancer Surgery | 3.600 | .058 | 1.000 |
| Total Health-Nathan T Thomas | 3.600 | .058 | 1.000 |
| The Women's Hospital-The London Clinic | .229 | .633 | 1.000 |
| The Women's Hospital-Buoy Health | .015 | .902 | 1.000 |
| The Women's Hospital-John Hopkins Medicine | .010 | .919 | 1.000 |
| The Women's Hospital-Net Doctor | .006 | .936 | 1.000 |
| The Women's Hospital-iqclinic | .439 | .508 | 1.000 |
| The Women's Hospital-Komen | 1.270 | .260 | 1.000 |
| The Women's Hospital-Breast cancer org | 1.023 | .312 | 1.000 |
| The Women's Hospital-Mayo Clinic | 1.233 | .267 | 1.000 |
| The Women's Hospital-Cleveland clinic | 1.714 | .190 | 1.000 |
| The Women's Hospital-Healthline | 5.114 | .024 | 1.000 |
| The Women's Hospital-Up to date | 2.057 | .151 | 1.000 |
| The Women's Hospital-Very Well Health | 6.146 | .013 | 1.000 |
| The Women's Hospital-Medical News Today | 1.497 | .221 | 1.000 |
| The Women's Hospital-Shape | 4.980 | .026 | 1.000 |
| The Women's Hospital-Stony Brook Medical Center | 5.714 | .017 | 1.000 |
| The Women's Hospital-Wikipedia | 16.785 | <.001 | .031 |
| The Women's Hospital-iheartpathology | .229 | .633 | 1.000 |
| The Women's Hospital-Medicine.net | 8.417 | .004 | 1.000 |
| The Women's Hospital-Breast Cancer Hub | 5.714 | .017 | 1.000 |
| The Women's Hospital-Gp notebook | 35.714 | <.001 | .000 |
| The Women's Hospital-Radiology info | 16.785 | <.001 | .031 |
| The Women's Hospital-Teach me surgery | 5.714 | .017 | 1.000 |
| The Women's Hospital-Specialist Breast Cancer Surgery | 5.714 | .017 | 1.000 |
| The Women's Hospital-Nathan T Thomas | 5.714 | .017 | 1.000 |
| The London Clinic-Buoy Health | .178 | .673 | 1.000 |
| The London Clinic-John Hopkins Medicine | .160 | .689 | 1.000 |
| The London Clinic-Net Doctor | .134 | .714 | 1.000 |
| The London Clinic-iqclinic | .160 | .689 | 1.000 |
| The London Clinic-Komen | .222 | .637 | 1.000 |
| The London Clinic-Breast cancer org | .182 | .670 | 1.000 |
| The London Clinic-Mayo Clinic | .172 | .679 | 1.000 |
| The London Clinic-Cleveland clinic | .240 | .624 | 1.000 |
| The London Clinic-Healthline | .185 | .667 | 1.000 |
| The London Clinic-Up to date | .400 | .527 | 1.000 |
| The London Clinic-Very Well Health | .222 | .637 | 1.000 |
| The London Clinic-Medical News Today | .160 | .689 | 1.000 |
| The London Clinic-Shape | .134 | .714 | 1.000 |
| The London Clinic-Stony Brook Medical Center | 3.600 | .058 | 1.000 |
| The London Clinic-Wikipedia | 1.862 | .172 | 1.000 |
| The London Clinic-iheartpathology | .400 | .527 | 1.000 |
| The London Clinic-Medicine.net | 1.879 | .170 | 1.000 |
| The London Clinic-Breast Cancer Hub | .400 | .527 | 1.000 |
| The London Clinic-Gp notebook | 2.057 | .151 | 1.000 |
| The London Clinic-Radiology info | 1.862 | .172 | 1.000 |
| The London Clinic-Teach me surgery | 3.600 | .058 | 1.000 |
| The London Clinic-Specialist Breast Cancer Surgery | 3.600 | .058 | 1.000 |
| The London Clinic-Nathan T Thomas | 3.600 | .058 | 1.000 |
| Buoy Health-John Hopkins Medicine | .000 | 1.000 | 1.000 |
| Buoy Health-Net Doctor | .000 | 1.000 | 1.000 |
| Buoy Health-iqclinic | .000 | 1.000 | 1.000 |
| Buoy Health-Komen | .110 | .740 | 1.000 |
| Buoy Health-Breast cancer org | .000 | 1.000 | 1.000 |
| Buoy Health-Mayo Clinic | .233 | .629 | 1.000 |
| Buoy Health-Cleveland clinic | .125 | .724 | 1.000 |
| Buoy Health-Healthline | 1.500 | .221 | 1.000 |
| Buoy Health-Up to date | .278 | .598 | 1.000 |
| Buoy Health-Very Well Health | 1.444 | .229 | 1.000 |
| Buoy Health-Medical News Today | .300 | .584 | 1.000 |
| Buoy Health-Shape | 4.500 | .034 | 1.000 |
| Buoy Health-Stony Brook Medical Center | 5.881 | .015 | 1.000 |
| Buoy Health-Wikipedia | 14.580 | <.001 | .100 |
| Buoy Health-iheartpathology | .278 | .598 | 1.000 |
| Buoy Health-Medicine.net | 5.000 | .025 | 1.000 |
| Buoy Health-Breast Cancer Hub | 2.179 | .140 | 1.000 |
| Buoy Health-Gp notebook | 26.923 | <.001 | .000 |
| Buoy Health-Radiology info | 14.580 | <.001 | .100 |
| Buoy Health-Teach me surgery | 5.881 | .015 | 1.000 |
| Buoy Health-Specialist Breast Cancer Surgery | 5.881 | .015 | 1.000 |
| Buoy Health-Nathan T Thomas | 5.881 | .015 | 1.000 |
| John Hopkins Medicine-Net Doctor | .000 | 1.000 | 1.000 |
| John Hopkins Medicine-iqclinic | .000 | 1.000 | 1.000 |
| John Hopkins Medicine-Komen | .685 | .408 | 1.000 |
| John Hopkins Medicine-Breast cancer org | .000 | 1.000 | 1.000 |
| John Hopkins Medicine-Mayo Clinic | .855 | .355 | 1.000 |
| John Hopkins Medicine-Cleveland clinic | 1.138 | .286 | 1.000 |
| John Hopkins Medicine-Healthline | .817 | .366 | 1.000 |
| John Hopkins Medicine-Up to date | 2.564 | .109 | 1.000 |
| John Hopkins Medicine-Very Well Health | .985 | .321 | 1.000 |
| John Hopkins Medicine-Medical News Today | 1.600 | .206 | 1.000 |
| John Hopkins Medicine-Shape | 2.400 | .121 | 1.000 |
| John Hopkins Medicine-Stony Brook Medical Center | 2.564 | .109 | 1.000 |
| John Hopkins Medicine-Wikipedia | 2.520 | .112 | 1.000 |
| John Hopkins Medicine-iheartpathology | .649 | .420 | 1.000 |
| John Hopkins Medicine-Medicine.net | 1.200 | .273 | 1.000 |
| John Hopkins Medicine-Breast Cancer Hub | 2.564 | .109 | 1.000 |
| John Hopkins Medicine-Gp notebook | 4.583 | .032 | 1.000 |
| John Hopkins Medicine-Radiology info | 10.080 | .001 | 1.000 |
| John Hopkins Medicine-Teach me surgery | 2.564 | .109 | 1.000 |
| John Hopkins Medicine-Specialist Breast Cancer Surgery | 2.564 | .109 | 1.000 |
| John Hopkins Medicine-Nathan T Thomas | 2.564 | .109 | 1.000 |
| Net Doctor-iqclinic | .000 | 1.000 | 1.000 |
| Net Doctor-Komen | .004 | .949 | 1.000 |
| Net Doctor-Breast cancer org | .000 | 1.000 | 1.000 |
| Net Doctor-Mayo Clinic | .000 | 1.000 | 1.000 |
| Net Doctor-Cleveland clinic | .011 | .915 | 1.000 |
| Net Doctor-Healthline | .000 | 1.000 | 1.000 |
| Net Doctor-Up to date | .134 | .714 | 1.000 |
| Net Doctor-Very Well Health | 1.782 | .182 | 1.000 |
| Net Doctor-Medical News Today | .600 | .439 | 1.000 |
| Net Doctor-Shape | 3.200 | .074 | 1.000 |
| Net Doctor-Stony Brook Medical Center | 3.348 | .067 | 1.000 |
| Net Doctor-Wikipedia | 4.320 | .038 | 1.000 |
| Net Doctor-iheartpathology | .536 | .464 | 1.000 |
| Net Doctor-Medicine.net | 4.500 | .034 | 1.000 |
| Net Doctor-Breast Cancer Hub | .536 | .464 | 1.000 |
| Net Doctor-Gp notebook | 4.294 | .038 | 1.000 |
| Net Doctor-Radiology info | 4.320 | .038 | 1.000 |
| Net Doctor-Teach me surgery | 3.348 | .067 | 1.000 |
| Net Doctor-Specialist Breast Cancer Surgery | 8.571 | .003 | 1.000 |
| Net Doctor-Nathan T Thomas | 3.348 | .067 | 1.000 |
| iqclinic-Komen | .007 | .934 | 1.000 |
| iqclinic-Breast cancer org | .000 | 1.000 | 1.000 |
| iqclinic-Mayo Clinic | .333 | .564 | 1.000 |
| iqclinic-Cleveland clinic | .218 | .641 | 1.000 |
| iqclinic-Healthline | .267 | .606 | 1.000 |
| iqclinic-Up to date | .361 | .548 | 1.000 |
| iqclinic-Very Well Health | .207 | .649 | 1.000 |
| iqclinic-Medical News Today | .400 | .527 | 1.000 |
| iqclinic-Shape | .600 | .439 | 1.000 |
| iqclinic-Stony Brook Medical Center | 2.564 | .109 | 1.000 |
| iqclinic-Wikipedia | 1.120 | .290 | 1.000 |
| iqclinic-iheartpathology | .361 | .548 | 1.000 |
| iqclinic-Medicine.net | 1.200 | .273 | 1.000 |
| iqclinic-Breast Cancer Hub | .361 | .548 | 1.000 |
| iqclinic-Gp notebook | 7.299 | .007 | 1.000 |
| iqclinic-Radiology info | 10.080 | .001 | 1.000 |
| iqclinic-Teach me surgery | 2.564 | .109 | 1.000 |
| iqclinic-Specialist Breast Cancer Surgery | 2.564 | .109 | 1.000 |
| iqclinic-Nathan T Thomas | 2.564 | .109 | 1.000 |
| Komen-Breast cancer org | .012 | .914 | 1.000 |
| Komen-Mayo Clinic | .320 | .572 | 1.000 |
| Komen-Cleveland clinic | .062 | .803 | 1.000 |
| Komen-Healthline | 1.680 | .195 | 1.000 |
| Komen-Up to date | .222 | .637 | 1.000 |
| Komen-Very Well Health | 2.178 | .140 | 1.000 |
| Komen-Medical News Today | .385 | .535 | 1.000 |
| Komen-Shape | 4.672 | .031 | 1.000 |
| Komen-Stony Brook Medical Center | 6.019 | .014 | 1.000 |
| Komen-Wikipedia | 10.258 | .001 | 1.000 |
| Komen-iheartpathology | .222 | .637 | 1.000 |
| Komen-Medicine.net | 5.178 | .023 | 1.000 |
| Komen-Breast Cancer Hub | 2.000 | .157 | 1.000 |
| Komen-Gp notebook | 18.337 | <.001 | .014 |
| Komen-Radiology info | 11.533 | <.001 | .507 |
| Komen-Teach me surgery | 6.019 | .014 | 1.000 |
| Komen-Specialist Breast Cancer Surgery | 6.019 | .014 | 1.000 |
| Komen-Nathan T Thomas | 6.019 | .014 | 1.000 |
| Breast cancer org-Mayo Clinic | .213 | .644 | 1.000 |
| Breast cancer org-Cleveland clinic | .107 | .744 | 1.000 |
| Breast cancer org-Healthline | 1.320 | .251 | 1.000 |
| Breast cancer org-Up to date | .262 | .609 | 1.000 |
| Breast cancer org-Very Well Health | 2.359 | .125 | 1.000 |
| Breast cancer org-Medical News Today | .463 | .496 | 1.000 |
| Breast cancer org-Shape | 4.320 | .038 | 1.000 |
| Breast cancer org-Stony Brook Medical Center | 5.704 | .017 | 1.000 |
| Breast cancer org-Wikipedia | 16.000 | <.001 | .047 |
| Breast cancer org-iheartpathology | .262 | .609 | 1.000 |
| Breast cancer org-Medicine.net | 8.820 | .003 | 1.000 |
| Breast cancer org-Breast Cancer Hub | 2.103 | .147 | 1.000 |
| Breast cancer org-Gp notebook | 36.502 | <.001 | .000 |
| Breast cancer org-Radiology info | 17.647 | <.001 | .020 |
| Breast cancer org-Teach me surgery | 5.704 | .017 | 1.000 |
| Breast cancer org-Specialist Breast Cancer Surgery | 5.704 | .017 | 1.000 |
| Breast cancer org-Nathan T Thomas | 5.704 | .017 | 1.000 |
| Mayo Clinic-Cleveland clinic | .155 | .694 | 1.000 |
| Mayo Clinic-Healthline | .200 | .655 | 1.000 |
| Mayo Clinic-Up to date | .305 | .581 | 1.000 |
| Mayo Clinic-Very Well Health | .720 | .396 | 1.000 |
| Mayo Clinic-Medical News Today | .333 | .564 | 1.000 |
| Mayo Clinic-Shape | 2.133 | .144 | 1.000 |
| Mayo Clinic-Stony Brook Medical Center | 2.307 | .129 | 1.000 |
| Mayo Clinic-Wikipedia | 5.333 | .021 | 1.000 |
| Mayo Clinic-iheartpathology | .305 | .581 | 1.000 |
| Mayo Clinic-Medicine.net | 3.733 | .053 | 1.000 |
| Mayo Clinic-Breast Cancer Hub | .305 | .581 | 1.000 |
| Mayo Clinic-Gp notebook | 11.349 | <.001 | .559 |
| Mayo Clinic-Radiology info | 7.680 | .006 | 1.000 |
| Mayo Clinic-Teach me surgery | 2.307 | .129 | 1.000 |
| Mayo Clinic-Specialist Breast Cancer Surgery | 6.176 | .013 | 1.000 |
| Mayo Clinic-Nathan T Thomas | 2.307 | .129 | 1.000 |
| Cleveland clinic-Healthline | .028 | .867 | 1.000 |
| Cleveland clinic-Up to date | .240 | .624 | 1.000 |
| Cleveland clinic-Very Well Health | .062 | .803 | 1.000 |
| Cleveland clinic-Medical News Today | .018 | .894 | 1.000 |
| Cleveland clinic-Shape | .011 | .915 | 1.000 |
| Cleveland clinic-Stony Brook Medical Center | 2.679 | .102 | 1.000 |
| Cleveland clinic-Wikipedia | 2.667 | .102 | 1.000 |
| Cleveland clinic-iheartpathology | .240 | .624 | 1.000 |
| Cleveland clinic-Medicine.net | 2.845 | .092 | 1.000 |
| Cleveland clinic-Breast Cancer Hub | .240 | .624 | 1.000 |
| Cleveland clinic-Gp notebook | 3.360 | .067 | 1.000 |
| Cleveland clinic-Radiology info | 4.507 | .034 | 1.000 |
| Cleveland clinic-Teach me surgery | 2.679 | .102 | 1.000 |
| Cleveland clinic-Specialist Breast Cancer Surgery | 6.000 | .014 | 1.000 |
| Cleveland clinic-Nathan T Thomas | 2.679 | .102 | 1.000 |
| Healthline-Up to date | .251 | .616 | 1.000 |
| Healthline-Very Well Health | .715 | .398 | 1.000 |
| Healthline-Medical News Today | .267 | .606 | 1.000 |
| Healthline-Shape | 4.200 | .040 | 1.000 |
| Healthline-Stony Brook Medical Center | 5.586 | .018 | 1.000 |
| Healthline-Wikipedia | 8.153 | .004 | 1.000 |
| Healthline-iheartpathology | .251 | .616 | 1.000 |
| Healthline-Medicine.net | 6.000 | .014 | 1.000 |
| Healthline-Breast Cancer Hub | .251 | .616 | 1.000 |
| Healthline-Gp notebook | 16.972 | <.001 | .028 |
| Healthline-Radiology info | 9.387 | .002 | 1.000 |
| Healthline-Teach me surgery | 5.586 | .018 | 1.000 |
| Healthline-Specialist Breast Cancer Surgery | 5.586 | .018 | 1.000 |
| Healthline-Nathan T Thomas | 2.266 | .132 | 1.000 |
| Up to date-Very Well Health | .222 | .637 | 1.000 |
| Up to date-Medical News Today | .160 | .689 | 1.000 |
| Up to date-Shape | .134 | .714 | 1.000 |
| Up to date-Stony Brook Medical Center | .400 | .527 | 1.000 |
| Up to date-Wikipedia | .182 | .670 | 1.000 |
| Up to date-iheartpathology | .400 | .527 | 1.000 |
| Up to date-Medicine.net | 1.879 | .170 | 1.000 |
| Up to date-Breast Cancer Hub | .400 | .527 | 1.000 |
| Up to date-Gp notebook | 2.057 | .151 | 1.000 |
| Up to date-Radiology info | 5.304 | .021 | 1.000 |
| Up to date-Teach me surgery | 3.600 | .058 | 1.000 |
| Up to date-Specialist Breast Cancer Surgery | 3.600 | .058 | 1.000 |
| Up to date-Nathan T Thomas | .400 | .527 | 1.000 |
| Very Well Health-Medical News Today | .385 | .535 | 1.000 |
| Very Well Health-Shape | .036 | .849 | 1.000 |
| Very Well Health-Stony Brook Medical Center | 2.000 | .157 | 1.000 |
| Very Well Health-Wikipedia | 1.799 | .180 | 1.000 |
| Very Well Health-iheartpathology | .222 | .637 | 1.000 |
| Very Well Health-Medicine.net | 1.977 | .160 | 1.000 |
| Very Well Health-Breast Cancer Hub | .222 | .637 | 1.000 |
| Very Well Health-Gp notebook | 8.584 | .003 | 1.000 |
| Very Well Health-Radiology info | 6.626 | .010 | 1.000 |
| Very Well Health-Teach me surgery | 2.000 | .157 | 1.000 |
| Very Well Health-Specialist Breast Cancer Surgery | 5.556 | .018 | 1.000 |
| Very Well Health-Nathan T Thomas | 2.000 | .157 | 1.000 |
| Medical News Today-Shape | .000 | 1.000 | 1.000 |
| Medical News Today-Stony Brook Medical Center | .361 | .548 | 1.000 |
| Medical News Today-Wikipedia | 1.120 | .290 | 1.000 |
| Medical News Today-iheartpathology | .361 | .548 | 1.000 |
| Medical News Today-Medicine.net | 1.200 | .273 | 1.000 |
| Medical News Today-Breast Cancer Hub | .361 | .548 | 1.000 |
| Medical News Today-Gp notebook | 2.497 | .114 | 1.000 |
| Medical News Today-Radiology info | 4.480 | .034 | 1.000 |
| Medical News Today-Teach me surgery | 2.564 | .109 | 1.000 |
| Medical News Today-Specialist Breast Cancer Surgery | 2.564 | .109 | 1.000 |
| Medical News Today-Nathan T Thomas | 2.564 | .109 | 1.000 |
| Shape-Stony Brook Medical Center | .134 | .714 | 1.000 |
| Shape-Wikipedia | .000 | 1.000 | 1.000 |
| Shape-iheartpathology | .134 | .714 | 1.000 |
| Shape-Medicine.net | .000 | 1.000 | 1.000 |
| Shape-Breast Cancer Hub | .000 | 1.000 | 1.000 |
| Shape-Gp notebook | .407 | .524 | 1.000 |
| Shape-Radiology info | .480 | .488 | 1.000 |
| Shape-Teach me surgery | .536 | .464 | 1.000 |
| Shape-Specialist Breast Cancer Surgery | .536 | .464 | 1.000 |
| Shape-Nathan T Thomas | .536 | .464 | 1.000 |
| Stony Brook Medical Center-Wikipedia | .182 | .670 | 1.000 |
| Stony Brook Medical Center-iheartpathology | .400 | .527 | 1.000 |
| Stony Brook Medical Center-Medicine.net | .178 | .673 | 1.000 |
| Stony Brook Medical Center-Breast Cancer Hub | .400 | .527 | 1.000 |
| Stony Brook Medical Center-Gp notebook | .229 | .633 | 1.000 |
| Stony Brook Medical Center-Radiology info | .182 | .670 | 1.000 |
| Stony Brook Medical Center-Teach me surgery | .400 | .527 | 1.000 |
| Stony Brook Medical Center-Specialist Breast Cancer Surgery | .400 | .527 | 1.000 |
| Stony Brook Medical Center-Nathan T Thomas | .400 | .527 | 1.000 |
| Wikipedia-iheartpathology | .262 | .609 | 1.000 |
| Wikipedia-Medicine.net | .180 | .671 | 1.000 |
| Wikipedia-Breast Cancer Hub | .262 | .609 | 1.000 |
| Wikipedia-Gp notebook | 1.423 | .233 | 1.000 |
| Wikipedia-Radiology info | 2.560 | .110 | 1.000 |
| Wikipedia-Teach me surgery | 2.103 | .147 | 1.000 |
| Wikipedia-Specialist Breast Cancer Surgery | 2.103 | .147 | 1.000 |
| Wikipedia-Nathan T Thomas | .262 | .609 | 1.000 |
| iheartpathology-Medicine.net | .178 | .673 | 1.000 |
| iheartpathology-Breast Cancer Hub | .400 | .527 | 1.000 |
| iheartpathology-Gp notebook | .229 | .633 | 1.000 |
| iheartpathology-Radiology info | 1.862 | .172 | 1.000 |
| iheartpathology-Teach me surgery | .400 | .527 | 1.000 |
| iheartpathology-Specialist Breast Cancer Surgery | .400 | .527 | 1.000 |
| iheartpathology-Nathan T Thomas | .400 | .527 | 1.000 |
| Medicine.net-Breast Cancer Hub | .278 | .598 | 1.000 |
| Medicine.net-Gp notebook | .115 | .734 | 1.000 |
| Medicine.net-Radiology info | 2.880 | .090 | 1.000 |
| Medicine.net-Teach me surgery | .278 | .598 | 1.000 |
| Medicine.net-Specialist Breast Cancer Surgery | 2.179 | .140 | 1.000 |
| Medicine.net-Nathan T Thomas | .278 | .598 | 1.000 |
| Breast Cancer Hub-Gp notebook | .229 | .633 | 1.000 |
| Breast Cancer Hub-Radiology info | 1.862 | .172 | 1.000 |
| Breast Cancer Hub-Teach me surgery | .400 | .527 | 1.000 |
| Breast Cancer Hub-Specialist Breast Cancer Surgery | .400 | .527 | 1.000 |
| Breast Cancer Hub-Nathan T Thomas | .400 | .527 | 1.000 |
| Gp notebook-Radiology info | 2.109 | .146 | 1.000 |
| Gp notebook-Teach me surgery | .229 | .633 | 1.000 |
| Gp notebook-Specialist Breast Cancer Surgery | .229 | .633 | 1.000 |
| Gp notebook-Nathan T Thomas | .229 | .633 | 1.000 |
| Radiology info-Teach me surgery | .262 | .609 | 1.000 |
| Radiology info-Specialist Breast Cancer Surgery | .262 | .609 | 1.000 |
| Radiology info-Nathan T Thomas | .262 | .609 | 1.000 |
| Teach me surgery-Specialist Breast Cancer Surgery | .400 | .527 | 1.000 |
| Teach me surgery-Nathan T Thomas | .400 | .527 | 1.000 |
| Specialist Breast Cancer Surgery-Nathan T Thomas | .400 | .527 | 1.000 |
| Each row tests the null hypothesis that the Sample 1 and Sample 2 distributions are the same.  Asymptotic significances (2-sided tests) are displayed. The significance level is .050. | | | |
| a. Significance values have been adjusted by the Bonferroni correction for multiple tests. | | | |
