# Supplementary material for: An evaluation of the readability and visual appearance of online patient resources for fibroadenoma
Source: PLoS One. 2022 Nov 18;17(11):e0277823. doi: 10.1371/journal.pone.0277823 (PMC9674151; doi:10.1371/journal.pone.0277823)
Supplement: Attachment — Submitted filename: Response to Reviewers HH18082022.docx [file pone.0277823.s002.docx]

15 August 2022

Dear Mr Donlan,

Thank you for reviewing our manuscript and the helpful review comments. We have now revised the manuscript based on these comments and have provided a response to each of the reviewer comments below.

We hope that you will now consider our manuscript suitable for publication.

Yours faithfully,

Hayley Hutchings (Professor of Health Services Research)

Journal Requirements:

When submitting your revision, we need you to address these additional requirements.

1. Please ensure that your manuscript meets PLOS ONE's style requirements, including those for file naming. The PLOS ONE style templates can be found at 
<https://journals.plos.org/plosone/s/file?id=wjVg/PLOSOne_formatting_sample_main_body.pdf> and 
<https://journals.plos.org/plosone/s/file?id=ba62/PLOSOne_formatting_sample_title_authors_affiliations.pdf>

**Response**: We have reformatted our paper based on PLOS ONE’s style requirements.

2. We note that you have indicated that data from this study are available upon request. PLOS only allows data to be available upon request if there are legal or ethical restrictions on sharing data publicly. For more information on unacceptable data access restrictions, please see <http://journals.plos.org/plosone/s/data-availability#loc-unacceptable-data-access-restrictions>.

In your revised cover letter, please address the following prompts:

a) If there are ethical or legal restrictions on sharing a de-identified data set, please explain them in detail (e.g., data contain potentially sensitive information, data are owned by a third-party organization, etc.) and who has imposed them (e.g., an ethics committee). Please also provide contact information for a data access committee, ethics committee, or other institutional body to which data requests may be sent.

b) If there are no restrictions, please upload the minimal anonymized data set necessary to replicate your study findings as either Supporting Information files or to a stable, public repository and provide us with the relevant URLs, DOIs, or accession numbers. For a list of acceptable repositories, please see <http://journals.plos.org/plosone/s/data-availability#loc-recommended-repositories>.

We will update your Data Availability statement on your behalf to reflect the information you provide.

**Response**: There are no restrictions in terms of data sharing. We have now deposited our data in the Zenodo opendata repository at Swansea University

(Available at: https://zenodo.org/record/6992835#.Yv_Bji7MI2x)

3. We note that you have stated that you will provide repository information for your data at acceptance. Should your manuscript be accepted for publication, we will hold it until you provide the relevant accession numbers or DOIs necessary to access your data. If you wish to make changes to your Data Availability statement, please describe these changes in your cover letter and we will update your Data Availability statement to reflect the information you provide.

**Response**: Data have now been deposited on Zenoda (available at: [An Evaluation of the Readability and Visual Appearance of Online Patient Resources for Fibroadenoma | Zenodo](https://zenodo.org/record/6992835#.YvpNw_jMKUk))

4. Please upload a copy of Figure 6, to which you refer in your text on page 11. If the figure is no longer to be included as part of the submission please remove all reference to it within the text.

**Response**: Apologies this is an error. All references to Table 6 have been removed. This table is now referred to as Table 4 in the text.

[Note: HTML markup is below. Please do not edit.]

Reviewers' comments:

Reviewer's Responses to Questions

**Reviewer comments**

Review Comments to the Author

Please use the space provided to explain your answers to the questions above. You may also include additional comments for the author, including concerns about dual publication, research ethics, or publication ethics. (Please upload your review as an attachment if it exceeds 20,000 characters)

Reviewer #1: Although the study is well executed and on an important topic, the sample size of 10 websites evaluated in 2020 calls into question the scope and strength, as well as the currency, of the authors' findings. Work that evaluated a greater number of websites covering more search phrases than the single "fibroadenoma," perhaps employing more sophisticated analytic techniques to account for greater sample size, or perhaps including resources in other languages, would perhaps be more appropriate for PLOS.

**Response**: Thank you for these helpful comments to improve the paper. We have now undertaken a more extensive search and analysis of the data:

1. We used three search engines- Google, Bing and Yahoo to identify relevant websites.
2. We expanded the search terms used with these three search engines:
   a. Fibroadenoma
   b. Breast lumps
   c. Non-cancerous breast lumps
   d. Benign breast lumps
   e. Benign breast lesions
3. We have now used more extensive assessment metrics to evaluate the readability of the 39 identified websites.

Reviewer #2: I would remove some redundant information and will make the introduction shortened. The introduction includes too detailed information about fibroadenoma that is not relevant to this research purpose, so I would more focus on why assessing the readability of online information for fibroadenoma is important.

**Response**: Thank you. We have now shortened the introduction and removed some redundant information. The Introduction is now focused on readability.

I think that two separate people did the readability assessment, compared the results, and resolved the conflicting results by conversation; however, this process is not included in the Methods. I would include such information in the Method.

**Response**: Thank you. This is correct- we have now included this information in the Methods.

In the discussion, I would include what type of support can be provided to increase the readability of information in a health care setting and/or community level. The current discussion focused on educating website developers but we may need to think what other level of setting can do to increase its readability.

**Response:** Thank you. We have now included information in the discussion about this.
